# Hospital Mortality Among Elderly Patients Admitted With Neurological Disorders Was Not Predicted by any Particular Diagnosis in a Tertiary Medical Center

**DOI:** 10.2174/1874205X01812010001

**Published:** 2018-01-22

**Authors:** Aroldo Bacellar, Telma Assis, Bruno B. Pedreira, Gersonita Costa, Osvaldo J.M. Nascimento

**Affiliations:** Hospital Sao Rafael, Department of Neurology Av Sao Rafael 2152, Sao Marcos, Salvador, BA, CEP 41235-190, Brazil

**Keywords:** Hospital mortality, Risk factors, Nervous system diseases, Comorbidity, Aged and inpatients

## Abstract

**Background:**

Neurological disorders (NDs) are associated with high hospital mortality. We aimed to identify predictors of hospital mortality among elderly inpatients with NDs.

**Methods:**

Patients aged ≥60 years admitted to the hospital between January 1, 2009 and December 31, 2010 with acute NDs, chronic NDs as underpinnings of acute clinical disorders, and neurological complications of other diseases were studied. We analyzed demographic data, NDs, and comorbidities as independent predictors of hospital mortality. Logistic regression was performed for multivariable analysis.

**Results:**

Overall, 1540 NDs and 2679 comorbidities were identified among 798 inpatients aged ≥ 60 years (mean 75.8±9.1). Of these, 54.5% were female. Diagnostic frequency of NDs ranged between 0.3% and 50.8%. Diagnostic frequency of comorbidities ranged from 5.6% to 84.5%. Comorbidities varied from 0 to 9 per patient (90% of patients had ≥2 comorbidities), mean 3.2±1.47(CI, 3.1-3.3). Patients with multimorbidities presented with a mean of 4.7±1.7 morbidities per patient. Each ND and comorbidity were associated with high hospital mortality, producing narrow ranges between the lowest and highest incidences of death (hospital mortality = 18%) (95% CI, 15%-21%). After multivariable analysis, advanced age (P<0.001) and low socioeconomic status (P=0.003) were recognized as predictors of mortality, totaling 9% of the variables associated with hospital mortality.

**Conclusion:**

Neither a particular ND nor an individual comorbidity predicted hospital mortality. Age and low socioeconomic class accounted for 9% of predictors. We suggest evaluating whether functional, cognitive, or comorbidity scores will improve the risk model of hospital mortality in elderly patients admitted with ND.

## 1 INTRODUCTION

Aging with multiple chronic disorders is associated with disability, and accounts for the majority of population deaths [1-3]. Neurological disorders (NDs) are common among the elderly, and cause a great burden to patients and healthcare systems, and their numbers are likely to greatly increase in the next few decades [4, 5]. A population-based study showed that not only neurological disorders but also their initial symptoms predict mortality in elderly patients [6]. In Salvador, Brazil, cerebrovascular disease represents the first cause of death with an annual mortality of 45 /100 000 inhabitants. This rate rises according to the age of the population, being 138/100 000 in persons aged 60-69 years, and 366 /100 000 in those aged 70 to 79 years [7]. Neurological disorders are severely disabling and patients are often admitted to tertiary care hospitals [4, 8, 9]. Moreover, acute NDs such as stroke, intensive-care-unit-acquired weakness, delirium, epilepsy and epileptic seizures are associated with high hospital mortality [10-14]. Although the trend towards hospital death due to dementia is reversing, two of five patients with dementia died in hospitals [15]. In addition, data linkage of hospital records and death certificate records nearly doubles the identification of deaths with dementia [16]. Furthermore, patients who suffer from NDs such as cerebrovascular disorders, Parkinson’s disease, dementia, muscular dystrophy, amyotrophic lateral sclerosis, myasthenia gravis and chronic polyneuropathies, usually die in hospital [17-19]. A two-year hospital mortality incidence of 18% (95% CI, 15%-21%) was found in elderly inpatients with NDs admitted to a tertiary medical center in Salvador, Brazil [20]. Predictors of hospital mortality studies covering total NDs are scarce, since previous studies have usually focused on single conditions. Hence, this study aimed to analyze demographics, NDs, and comorbidities in elderly patients admitted with NDs, to identify predictors of hospital mortality in this particular population.

## 2 METHODS

### 2.1 Population

Patients aged 60 years or more consecutively admitted to the Hospital São Rafael (HSR) in Salvador between January 1, 2009 and December 31, 2010 were enrolled. The HSR is a general tertiary teaching hospital, which admits patients who are clients of several private health services (supplementary health systems) and users of the Brazilian Unified Public Health System (SUS-users). The HSR uses electronic medical records, which makes it easy to find patients with NDs in discharge lists, as well as in registries of neurological procedures. This hospital remunerates physicians using pay-for- performance. Every physician in the HSR uses billing codes to register hospital procedures such as visits, consultations, surgeries, thrombolytic therapy and other medical practices. The Information Technology Department at this hospital (IT-HSR) searches billing codes in order to send invoices of the medical procedures to health sponsors and for paying physicians. Further information regarding the HSR and the method for capturing patients, including details regarding ND diagnostic assessments, can be found in our previously published study of the same population [20].

We enrolled elderly inpatients with NDs to this study using the following methods: 1. IT-HSR searches of NDs listed in discharge summaries; 2. IT-HSR identification of registered neurological procedures; 3. After the IT-HSR selection (obtained from the discharge list and neurological procedures information), the authors carefully examined all of the written patient health records. This method assured a corroborated data bank, and captured all inpatients with NDs that might have influenced hospital mortality. Furthermore, this process recognized patients with more than one ND.

We selected elderly inpatients with neurological symptoms based on the following inclusion criteria: (1) patients admitted with NDs to be treated by neurologists; (2) patients admitted with clinical disorders owing to an underlying chronic neurological disease that needed care or follow-on therapy by a neurologist; (3) medical, surgical, or neurosurgical patients who suffered neurological complications during their hospital stay and required consultation with a neurologist.

Exclusion criteria were as follows: (1) patients who were admitted by or consulted with a neurologist, but had no neurological disorder; (2) patients whose medical records lacked important data; (3) patients with acute trauma, subarachnoid hemorrhage, central nervous system tumor, or other neurosurgical disorder who were referred to a neurosurgeon; (4) patients who underwent neurological consultation for diagnosis of brain death due to head trauma, cardiovascular arrest, or other condition out of the scope of neurologists due to neurosurgical or oncological events; (5) patients transferred to another hospital without an established diagnosis.

### 2.2 Demographics

Influences of age, gender, marital status, and socioeconomic level on hospital mortality were considered. Differences of hospital mortality concerning socioeconomic status were estimated comparing SUS-users with patients who were clients of private health services. Elderly patients who belong to families categorized according to Brazilian social economic strata criteria [21], as B2 or lower socioeconomic classes (average monthly income equal or less than 4852 Brazilian Reals, that is the equivalent to US $ 1475) used SUS [22]. Whereas elders from the Brazilian middle (class B1 with average family monthly income of R$ 9254 or US $ 2813) and upper social classes prefered to pay for private health services [21-23].

### 2.3 Neurological Disorders

Diagnostic criteria for NDs were based on the Tenth Revision of the International Statistical Classification of Diseases and Related Health Problems (ICD-10) [24]. Disease frequency and hospital mortality rates of the following neurological disorders were analyzed: cerebrovascular disorders represented by ischemic stroke, transient ischemic attack, and spontaneous brain hemorrhage (excluding subarachnoid hemorrhage); epilepsy and acute symptomatic epileptic seizures; movement disorders including hyperkinetic disorders (Huntington's chorea was classified as a movement disorder), Parkinson’s disease (PD) and parkinsonism (but not cases of PD dementia or Lewy bodies dementia) ; neuromuscular disorders; central nervous system infections; headache; syncope or near-syncope; central nervous system (CNS) toxic and metabolic disorders including alcoholism and other toxic or metabolic encephalopathies as well as acute neurological complications due to water- electrolyte balance disturbances, and brain injury as a sequel to cardiovascular arrest. In addition, CNS neoplasms were included if patients under the care of an oncologist or neurosurgeon had consulted a neurologist for clinical treatment of NDs, such as epilepsy, headache, or cognitive disorders, and neurocognitive disorders were also included if cases of delirium and cases of dementia were diagnosed based on DSM-IV criteria [25]. Dementia and delirium were independently analyzed. Degenerative dementia such as Alzheimer disease, fronto-temporal dementia, Lewy bodies dementia, as well as vascular dementia were analyzed together since they do not have significant differences in short-term mortality rates in the elderly [26].

### 2.4 Comorbidities

Comorbidities were recognized by the following criteria. Hypertension was Diagnosed According to the Criteria of the Joint National committee on the Prevention, Detection, Evaluation and Treatment of High Blood Pressure [27]. [2] *Dyslipidemias* were defined according to the recommendations of the National Cholesterol Education Program Expert Panel on Detection, Evaluation and Treatment of High Blood Cholesterol in Adults (Adult Treatment Panel III) and the results of recent clinical trials [28, 29]. (3) *Diabetes mellitus* was diagnosed based on the follow-up report of The Expert Committee on Diagnosis and Classification of Diabetes Mellitus [30]. (4) *Clinical disorders* were classified according to ICD-10, and included a) infections, represented not only by patients who were admitted with infections, but also patients with neurological symptoms who experienced infections during their period of hospitalization, b) neoplasms, c) chronic and acute respiratory system diseases, d) musculoskeletal diseases, e) genitourinary disorders, f) digestive disorders, g) endocrine and metabolic disorders, including fluid and electrolyte disturbances (hepatic insufficiency was considered a metabolic rather than a digestive disorder), h) circulatory system disorders (cardiac and peripheral vascular disorders) excluding patients with cerebrovascular diseases, and i) psychiatric disorders were diagnosed based on DSM-IV criteria [25]. These disorders were only classified as comorbidities of NDs, since HSR does not admit patients with a primary psychiatric diagnosis; therefore most of the disorders were anxiety, depression, and bipolar disorder. (5) Patient multimorbidity signified patients suffering from two or more morbidities.

### 2.5 Predictors of Hospital Mortality

Patients were dichotomized between those who died in hospital and inpatients who remained alive. Univariate analysis of hospital mortality risk, including all patient characteristics, was performed; a multivariable analysis of selected variables was executed thereafter.

### 2.6 Statistics

Quantitative variables with normal distribution were reported as their mean and standard deviation and for variables with non-normal distribution by their median and interquartile interval.

Normal variables were identified by graphic analysis and the Shapiro-Wilk test. Categorical variables were reported by frequencies and percentages.

Bivariate comparisons between groups were performed with Student *t* test for numerical variables with normal distribution and by the Mann-Whitney test for those with non- normal distribution. Categorical variables were compared with Pearson's chi-square or Fisher's test when necessary.

Independent variables were considered for multivariable analysis if identified as a biological plausibility associated with main study hypotheses, and when bivariate tests showed a P value <0.25 according to the algorithm proposed by Hosmer and Lemeshow [31]. The number of variables was conservatively calculated (EPV= m/10). Hierarchic logistic regressions were sequentially performed in blocks to increment prediction power of a variable power model; sociodemographic characteristics were selected as initial independent variables (first block), followed by comorbidities (second block) and NDs (third block).

Full model fit was reported, including no significant variables by Wald statistics.

### 2.7 Ethics

The HSR Ethical Committee for Research approved this study (number 8/11) on 25 August 2011. The HSR Ethical Committee for Research was accredited by the National Committee for Ethics in Research, according to the Brazilian Operational Manual for Ethics Committees in Research.

## 3 RESULTS

### 3.1 Patient Demographics

During the study period 798 elderly inpatients with NDs that needed neurological care were admitted to HSR. The age of these individuals had a normal distribution with a mean of 75.8 ± 9.1 years and median of 76 years. The interquartile interval ranged from 68 years (25 percentile) to 82 years (percentile 75). Women formed 55% of this population. This geriatric population consisted of 464 (58%) married individuals, and 713 (89%) were patients of private health services.

### 3.2 Elderly Inpatient Hospital Mortality Rate

The hospital mortality rate for elderly inpatients with NDs was 18% (95% CI, 15%-21%).

#### 3.2.1 Neurological Disorders and Comorbidities: Frequency and Association with Hospital Mortality

Among our older adult inpatient population, 555 (70%) had primary NDs diagnosed by neurologists and identified in discharge summaries. The remaining 243 (30%) patients were admitted with clinical complications of underlying neurological chronic NDs or patients who developed neurological complications during their stay in the hospital. The latter were captured by IT-HSR examination of physician's billing codes. Overall, 312 (39%) patients were affected by more than one ND, totaling 668 additional NDs that were classified as neurological comorbidities in this population. Therefore, we found 1154 NDs among 798 elderly inpatients, which represented a mean NDs of 1.32±0.91 (95% CI, 1.23 -1.38), with a range of 1-5 NDs per patient. Moreover, we observed a wide range of diagnostic frequency of the NDs (0.3% to 50.8%), and cerebrovascular disease was the most common (50.8%). This finding contrasted with a slight variation (15% to 20%) between individual ND’s hospital mortality rate; except for patients admitted with headache and syncope (11%), Fig. (**1**). This study captured 2679 comorbidities involving 90% of patients with two or more comorbidities, constituting a mean of 3.35±1.51 (95% CI, 3.25-3.47), and a range of 0-9 comorbidities per patient. We found a wide range of diagnostic frequency for comorbidities (5.6% to 84.5%), and hypertension (84.5%) and diabetes (57.5%) were the most common. However, the comorbidities’ hospital mortality rates only varied from 15% to 27%, Fig. (**2**). Overall, for patient multimorbidity (NDs + comorbidities) the mean was 4.7±1.7 morbidities per patient, median 5.0, ranging from 1 to 11 morbidities.

### 3.3 Patient’s Characteristics Associated with Hospital Mortality

After univariate analysis, patient age (p<0.001), being an SUS-user (p=0.003), infection (p=0.011), and respiratory system disorder (p=0.024), were demographics and clinical characteristics associated with hospital mortality, in contrast to syncope and headache, which predicted lower risk for hospital mortality (p<0.05), (Tables **1** and **2**).

### 3.4 Multivariable Analysis

Table (**3**) shows summary statistics including a measure of relative quality Akaike information criterion); an estimation of how much the model explains the outcome (Nagelkerke R^2^); an omnibus test of model coefficients (p value), and goodness of fit for logistic regression model (Hosmer-Lemeshow test). Table (**4**) shows the adjusted odds ratio (Exp b) with a 95% confidence interval for the three blocks as well as p-values of variable coefficients according to Wald statistics. After the final multivariable model, age and being an SUS-user were identified as independent risks for hospital mortality. However, neither neurological disorders nor their comorbidities predicted hospital mortality in this particular population. Additionally, patients admitted with syncope had a statistically significant lower risk of hospital mortality in this sample. Identified risks of hospital mortality in this population accounted for 9% of predictors (Nagelkerke R^2^), (Table **3**).

Fig. (**3**) shows standard receiver operating characteristic (ROC) and analysis of the area under the curve, which quantified power of prediction for this model demonstrating a statistically significant result.

## 4 DISCUSSION

During a two-year period all elderly patients admitted with ND diagnosis as the primary diagnosis on discharge, and also as a comorbidity or complication of another disorder were enrolled for this study. This method allowed good accuracy regarding rates of disease frequency and hospital mortality of all NDs and their comorbidities that could influence hospital mortality for elderly patients admitted to HSR. The studied population consisted of aged inpatients, 25% of them being older than 81years of age, which reflected the Brazilian socioeconomic middle and upper class aging phenomena. Age and being a SUS-user were associated with higher mortality rates. Although, the SUS has improved medical care for the low-income population, it is still far from optimal [32]. Although created to be a universal health system for the entire Brazilian population, the SUS has been chronically underfunded and poorly structured [33]. For this reason local health authorities have long-term agreements with some well-equipped non-profit foundations to increase the numbers of public hospital beds. Hence, HSR allows 30% of hospital beds to be used by SUS-users. We highlight that elderly SUS-users had a statistically significant much higher hospital mortality rate than their counterparts using private health services. As mentioned previously, SUS-users belong to the Brazilian lower socioeconomic stratum. We suppose that higher hospital mortality among SUS-users is due to their previous precarious health conditions because of the primary healthcare inequality that occurs, particularly in northeastern municipalities of Brazil [23, 34, 35].

Notwithstanding that the frequency of NDs and comorbidities had a wide range, hospital mortality rates for all NDs as well as for their comorbidities were uniformly high. These findings resulted in a narrow range between the lowest and highest rate of hospital mortality. Our results implied that all elderly inpatients with NDs were seriously ill, whatever their neurological diagnosis or comorbidity, as all NDs had a similar high rate of hospital mortality. This was surprising, because at first we expected that cerebrovascular diseases, delirium, and epilepsy as well as comorbidities in the top rank of hospital mortality, such as infections and respiratory and cardiovascular disorders would be predictors of death in this population [12, 13, 36, 37]. Therefore, although revealing a high hospital mortality rate in this population, this study was unable to identify predictors of hospital mortality among NDs or among comorbidities. We emphasize that the population under study included very old inpatients with NDs, and patient multimorbidities were more the rule than the exception. Patient multimorbidity in elderly inpatients is associated with disability, functional decline, and poor outcome [38]. Our results suggest that for identifying predictors of hospital mortality in elderly inpatients with NDs, individual characteristics might be more relevant than neurological diagnosis. The exception was patients admitted for syncope who were independently associated with lower risk of hospital mortality in this sample. Syncope is very common in emergency rooms, caused by several disorder etiologies including neurocardiovascular instability syndrome, as well as cardiac diseases associated with intermediate-risk for mortality in patients aged ≥ 50 years; however overall, syncope has a relatively low hospital mortality rate [39, 40].

According to Nagelkerke R^2^ statistics, aging and being a SUS-user (citizen of low socioeconomic status) could explain only 9% of variables related to hospital mortality. Nonetheless other variables not included in this analysis such as patient multimorbidity, scores of functional and cognitive status, polypharmacy, and severity of disease were already identified as predictors of hospital mortality in other geriatric inpatients [38, 41, 42]. Hence, we cogitate whether scores of comorbidity, functional, and cognitive status should be evaluated and whether they will improve our model of risk for hospital mortality in elderly inpatients with NDs.

Limitations of this study should be noted because it was a retrospective survey carried out in a single center. However, the data obtained were powerful because we used two approaches to capture all NDs that might influence elderly patient hospital mortality, that is, examining the discharge lists and searching the neurological procedure records. Moreover, after selection, the authors carefully reviewed all patient written health records, which assured quality of data and found a relevant patient multimorbidity.

## CONCLUSION

Overall, although associated with high rates of mortality, neither an ND nor a particular comorbidity was recognized as an independent predictor of hospital mortality in the studied population. Age and low socioeconomic strata explained 9% of variables associated with hospital mortality. Therefore, we recommend evaluating whether scores of comorbidity, functional performance, and scores of cognitive status will improve the model of risk for hospital mortality in elderly patients admitted with neurological disorders.

## Figures and Tables

**Fig. (1):**
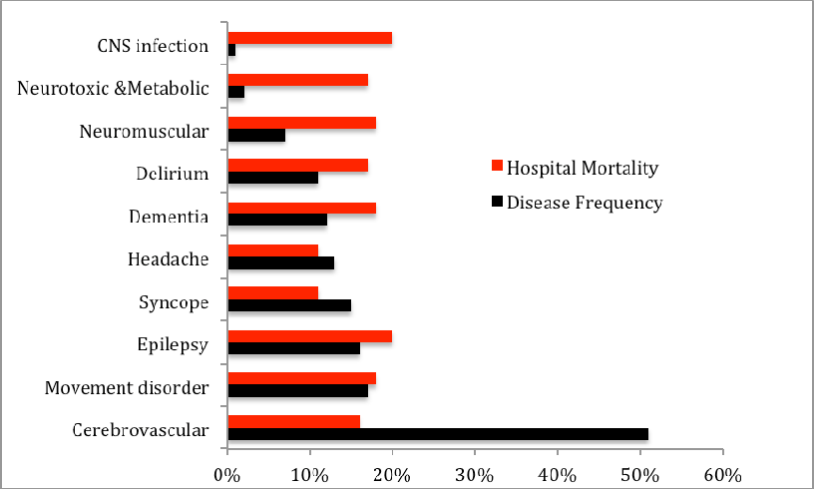
Nervous system disorder frequency and hospital mortality rate.

**Fig. (2):**
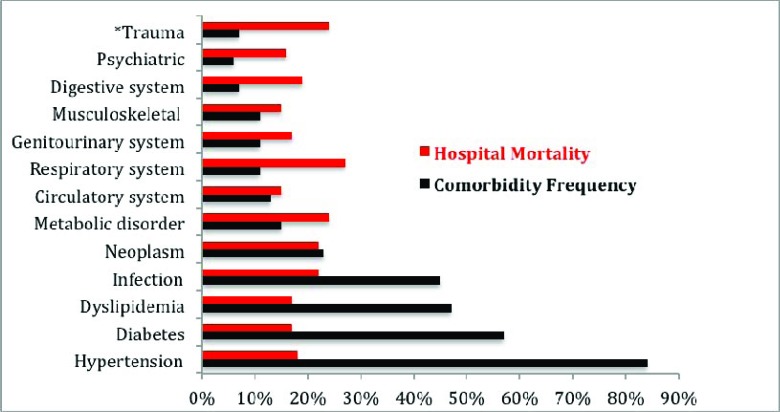
Comorbidities frequency and hospital mortality rate.

**Fig. (3):**
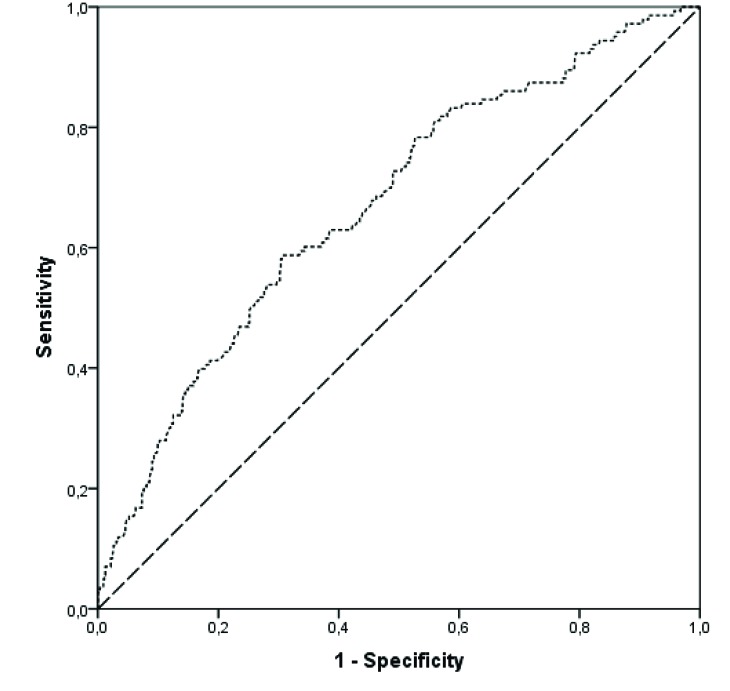
Area under the ROC (AUROC) = 0.671; IC 95%: 0.621 – 0.719; p < 0.001.

**Table 1:** Univariate Analysis of Demographics and Clinical Characteristics of Patients Related to Hospital Mortality.

**Characteristic**	**Total Patient Numbers** **N=798**	**Mortality**		**p value**
		**No** **n= 655**	**Yes** **n =143**	
Age	75.8 ± 9.1	75.1 ± 9.0	78.7 ± 9.2	<0.001
Female Sex	435 (54.5)	354 (54.0)	81 (56.6)	0.572
Married	464 (58.1)	384 (58.6)	80 (55.9)	0.556
*SUS user	85 (10.7)	60 (9.2)	25 (17.5)	0.003
Cerebrovascular disorder	405 (50.8)	342 (52.2)	63 (44.1)	0.077
Movement disorder	139 (17.4)	112 (17.1)	27 (18.9)	0.611
Epilepsy	127 (15.9)	102 (15.6)	25 (17.5)	0.572
Syncope	122 (15.3)	109(16.6)	13 (9.1)	0.023
Headache	102 (12.8)	91 (13.9)	11 (7.7)	0.044
Dementia	96(12.0)	79(12.1)	17(11.9)	0.954
Delirium	86(10.8)	71(10.8)	15(10.5)	0.903
Neuromuscular	55 (6.9)	45 (6.9)	10 (7.0)	0.958
Neurotoxic & Metabolic CNS infection	12 (1.5) 5 (0.6)	10 (1.5) 4 (0.6)	2 (1.4) 1 (0.7)	0.999 0.903
Brain injury	3 (0.4)	3 (0.5)	0 (0.0)	0.999
CNS neoplasm	2 (0.3)	2 (0.3)	0 (0.0)	0.999

Data are presented as total patient numbers and percentages within parentheses (%). *SUS user = users of Unified Brazilian Public Health System (**S**istema **Ú**nico de **S**aúde). CNS = central nervous system.

**Table 2:** Univariate Analysis of Comorbidity Characteristics According to Hospital Mortality.

**Comorbidity**	**Total Patient Numbers** **(N=798)**	**Mortality**		**p-value**	
		**No** **(n = 655)**	**Yes** **(n = 143)**		
Hypertension	674 (84.5)	550 (84.0)	124 (86.7)	0.412	
Diabetes	459 (57.5)	381 (58.2)	78 (54.5)	0.427	
Dyslipidemia	363 (47.4)	305 (48.6)	58 (42.0)	0.164	
Infection	359 (45.0)	281 (42.9)	78 (54.5)	0.011	
Neoplasm	181 (22.7)	141 (21.5)	40 (28.0)	0.095	
Endocrine & Metabolic disorder	122 (15.3)	93 (14,2)	29 (20.3)	0.067	
Circulatory system	102 (12.8)	87 (13.3)	15 (10.5)	0.409	
Genitourinary system	92 (11.5)	76 (11.6)	16 (11.2)	0.888	
Respiratory system	86 (10.8)	63 (9.6)	23 (16.1)	0.024	
Musculoskeletal	86 (10.8)	73 (11.1)	13 (9.1)	0.473	
Digestive system	57 (7.1)	46 (7.0)	11 (7.7)	0.778	
Trauma related	53 (6.6	40 (6.1)	13 (9.1)	0.194	
Psychiatric	45(5.6)	38(5.8)	7(4.9)	0.670	

Data are presented as total patient numbers and percentages within parentheses (%).

**Table 3:** Summary Statistics for Models According to Variable Blocks.

	**1^st^ Block**	**2^nd^ Block**	**3^rd^ Block**	**^b^**
*AIC	730.7	729.1	725.2	
Nagelkerke R^2^	0.056	0.071	0.090	
Omnibus Test of Model Coefficients^a^ (p value)	< 0.001	0.055	0.007	
Hosmer-Lemeshow test (p value)	0.146	0.439	0.312	

*AIC = Akaike Information Criterion.

**Table 4:** Multivariable Analysis: Adjusted Odds Ratio and Final P Value According to Wald Statistics.

	**1^st^ Block** **(Demographics)**	**2^nd^ Block** **(Comorbidities)**	**3^rd^ Block** **(Neurological Disorders)**	**Coefficient p value in final model** **(Wald statistics)**
	Adjusted Odds Ratio and 95% Confidence Interval			
Female Sex (Reference: Male)	1.103 (0.758 – 1.604)	1.140 (0.781 - 1.663)	1.142 (0.781 - 1.670)	0.493
Age (per year)	1.046 (1.026 – 1.068)	1.044 (1.023 - 1.066)	1.043 (1.022 - 1.065)	<0.001
SUS-user.	2.425 (1.438 – 4.088)	2.168 (1.256 - 3,.741)	2.124 (1.226 - 3.678)	0.007
Neoplasm	-	1.228 (0.791 - 1,.908)	1.222 (0.784 - 1.902)	0.376
Respiratory	-	1.481 (0.866 - 2.532)	1.409 (0.822 - 2.416)	0.213
Infection	-	1.447 (0.994 - 2.105)	1.305 (0.891 -– 1.913)	0.172
Cerebrovascular	-	-	0.712 (0.488 – 1.039)	0.079
Headache	-	-	0.530 (0.270 – 1.042)	0.066
Syncope	-	-	0.528 (0.282 – 0.988)	0.046

^a^ p values by Block. All models presented p < 0.001

## LIST OF ABBRIVIATIONS

AIC: Akaike information criterion
CNS: Central nervous system
HSR: Hospital Sao Rafael
IT-HSR: Information Technology of HSR
NDs: Neurological disorders
PD: Parkinson’s disease
ROC: Receiver operating characteristic
SUS: Brazilian unified public health system

## AVAILABILITY OF DATA AND MATERIALS

Datasets used and/or analyzed in this study are available from the corresponding author on reasonable request.

## AUTHOR DETAILS

1. Chairperson of The Neurology Department, Hospital Sao Rafael, Salvador-BA-BR
2. Neurologists of The Neurology Department, Hospital Sao Rafael
3. Head of Neurology Department at Fluminense Federal University, Niteroi, RJ-BR
